# Frontal dysconnectivity in 22q11.2 deletion syndrome: an atlas-based functional connectivity analysis

**DOI:** 10.1186/s12993-018-0134-y

**Published:** 2018-01-20

**Authors:** Leah M. Mattiaccio, Ioana L. Coman, Carlie A. Thompson, Wanda P. Fremont, Kevin M. Antshel, Wendy R. Kates

**Affiliations:** 1 Department of Psychiatry and Behavioral Sciences, State University of New York Upstate Medical University, 750 East Adams Street, Syracuse, NY USA; 2 Department of Computer Science, State University of New York at Oswego, Oswego, NY USA; 3 Department of Psychology, Syracuse University, Syracuse, NY 13210 USA

**Keywords:** Functional connectivity, Connectives, Frontal lobe dysconnectivity, Velo-cardio-facial syndrome, 22q11.2 deletion syndrome, Schizophrenia

## Abstract

**Background:**

22q11.2 deletion syndrome (22q11DS) is a neurodevelopmental syndrome associated with deficits in cognitive and emotional processing. This syndrome represents one of the highest risk factors for the development of schizophrenia. Previous studies of functional connectivity (FC) in 22q11DS report aberrant connectivity patterns in large-scale networks that are associated with the development of psychotic symptoms.

**Methods:**

In this study, we performed a functional connectivity analysis using the CONN toolbox to test for differential connectivity patterns between 54 individuals with 22q11DS and 30 healthy controls, between the ages of 17–25 years old. We mapped resting-state fMRI data onto 68 atlas-based regions of interest (ROIs) generated by the Desikan-Killany atlas in FreeSurfer, resulting in 2278 ROI-to-ROI connections for which we determined total linear temporal associations between each. Within the group with 22q11DS only, we further tested the association between prodromal symptoms of psychosis and FC.

**Results:**

We observed that relative to controls, individuals with 22q11DS displayed increased FC in lobar networks involving the frontal–frontal, frontal–parietal, and frontal–occipital ROIs. In contrast, FC between ROIs in the parietal–temporal and occipital lobes was reduced in the 22q11DS group relative to healthy controls. Moreover, positive psychotic symptoms were positively associated with increased functional connections between the left precuneus and right superior frontal gyrus, as well as reduced functional connectivity between the bilateral pericalcarine. Positive symptoms were negatively associated with increased functional connectivity between the right pericalcarine and right postcentral gyrus.

**Conclusions:**

Our results suggest that functional organization may be altered in 22q11DS, leading to disruption in connectivity between frontal and other lobar substructures, and potentially increasing risk for prodromal psychosis.

**Electronic supplementary material:**

The online version of this article (10.1186/s12993-018-0134-y) contains supplementary material, which is available to authorized users.

## Background

Chromosome 22q11.2 deletion syndrome (22q11DS) is caused by a microdeletion of approximately 50 genes on one copy of the q11.2 band of chromosome 22. Youth with the syndrome typically present with physical anomalies, cognitive impairments, and behavioral disorders [1, 2]. During adolescence and young adulthood, approximately 30–40% of individuals with 22q11DS develop a psychotic illness, usually schizophrenia [3–5]. This represents a significant increase over the risk for schizophrenia in the general population [6]. The neurobiological mechanisms underlying this increased risk for schizophrenia in individuals with 22q11DS are not well-understood.

Converging evidence supports the notion that idiopathic (non-syndromal) schizophrenia is a disorder of functional and structural dysconnectivity [7–11]. Studies of functional connectivity point to a preponderance of anomalies in frontal–temporal connectivity [12, 13], although frontal–parietal and frontal–occipital connections have also been implicated [14, 15]. Moreover, abnormalities have been observed in several large-scale, functional networks, including the default mode network, the salience network and the central executive network [16–18].

Although studies examining functional dysconnectivity in 22q11DS are much fewer in number, the findings are consistent with studies of idiopathic schizophrenia [19]. Results of these studies indicate anomalous connectivity in frontal lobe connections [20] and parieto–occipital connections [20–22]. Decreases in functional connectivity have also been observed, in partially overlapping samples, in the default mode [23–26], salience [24] and frontal–parietal networks [22, 24]. In a modularity analysis of overall functional network organization, Scariati and colleagues [27] observed increased modular segregation across superior parietal, frontal and inferior temporal lobes in individuals with 22q11DS. Associations between anomalous functional connectivity in 22q11DS and increased symptoms of psychosis have been observed in most [20, 22, 24], but not all studies [25].

To our knowledge, two studies by Scariati and colleagues [20, 27] have conducted a functional connectivity analysis of atlas-based, ROI-to-ROI structural connections in 22q11DS. Scariati and colleagues first reported widespread functional connectivity in individuals with 22q11DS, primarily affecting frontal and temporal lobe regions. In a more recent study [27], they focused on age differences by examining connectivity in a sample of 9–30 year-old individuals with 22q11DS that were divided into two age groups (groups split at 18 years old) for subanalyses. In both age groups, alterations of modular communities were found to affect the anterior cingulate cortex and parieto-occipital processing regions. However, in adults with 22q11DS, they observed nontypical modularity partition of the dorsolateral prefrontal cortex.

Here, we conduct an atlas-based functional connectivity analysis of ROI-to-ROI connections in individuals with 22q11DS who are specifically between the ages of 18 and 24 years, a time-frame that poses the greatest risk for developing psychotic illness. In this ROI-to-ROI based approach, we sought to assess connectivity patterns by matching an anatomical atlas to each subject’s own fMRI space. The methodological advantage of this approach is that data were not normalized to a standard template, thus obviating potentially problematic effects of warping the brain. Conceptually, a subject-specific, atlas-based approach can yield additional data about the functional architecture and organization of the brain [28, 29]. Moreover, the use of atlas-based ROIs provides a common framework to increase reproducibility across studies, and can be incorporated for use in multimodal studies. In order to implement this approach, we applied the functional connectivity toolbox, CONN [28–30], which has shown a high degree of interscan reliability [28] and has demonstrated disease-relevant functional connections between anatomically defined regions of the brain [30]. We hypothesized that ROI-to-ROI connectivity between sublobar frontal–parietal gyri, and frontal–temporal gyri would be anomalous in individuals with 22q11DS relative to controls, and that aberrant connectivity would be associated with symptoms of psychosis.

## Methods

### Participants

Data were acquired from a large-scale longitudinal study of risk factors for psychosis in 22q11DS conducted at SUNY Upstate Medical University, Syracuse, NY. Our sample consisted of 84 participants: 54 with 22q11DS (30 males; mean age 20.98, SD 2.35) and 30 controls (16 males; mean age 20.97, SD 1.46). The control sample consisted of 12 healthy siblings of individuals with 22q11DS, and 18 community controls. Since siblings and community controls did not differ in either demographic variables or measures of functional connectivity (Additional file 1), they were combined into one control group. A previous publication included 39 of the 54 (72.2%) participants with 22q11DS in the current report, which tested differential connectivity in resting-state networks utilizing independent component analysis and associations with psychiatric and neurocognitive functioning [22]. Additionally, a recent publication including a partially overlapping sample of the 22q11DS group in this report demonstrated hypoconnectivity as a classifier in the identification of 22q11DS versus control groups [24].

Diagnosis of 22q11DS was confirmed by fluorescence in situ hybridization (FISH). Recruitment details have been described previously [31]. Briefly, exclusion criteria included seizure disorder, fetal exposure to alcohol or drugs, parent-reported elevated lead levels or birth weight under 2500 g, loss of consciousness lasting longer than 15 min, paramagnetic implants, or orthodontic braces. Potential controls with a personal or family history of schizophrenia or bipolar disorder were also excluded [31]. Since data for the current report were taken from a longitudinal study, control participants who had presented with an anxiety disorder and/or depression at the first timepoint were excluded. However, the current report depicts data from the last (fourth) timepoint, and controls that subsequently developed an anxiety disorder or depression in the longitudinal study were included. Controls with ADHD or a learning disability were not excluded at any timepoint in the study to maximize comparability to higher functioning participants in the 22q11DS group. Of the 54 participants, 22 were being treated with one or more antidepressant, antianxiety, antipsychotic, or stimulant medications at the time of their scan. Three controls were being treated with either a stimulant and/or antidepressant/antianxiety medication. Details of the samples can be found in Table 1.

**Table 1:** Demographic and psychiatric data

	22q11DS N = 54	Controls N = 30	p value
Age^a^	20.98 (2.35)	20.97 (1.46)	0.990
Gender (male, %)	30 (55.6%)	16 (53.3%)	0.847
Full scale IQ^a^ *Psychiatric diagnosis, n (%)*	74.41 (12.0)	109.47 (16.02)	< 0.001
Psychotic disorder	5 (9.26%)	0 (0%)	0.024
ADHD	8 (14.81%)	5 (16.67%)	0.825
Anxiety disorder	11 (20.37%)	4 (13.33%)	0.426
Mood disorder *Current medication, n (%)*	7 (12.96%)	1 (6.25%)	0.094
Antipsychotic/mood stabilizer	8 (14.81%)	0 (0%)	0.004
Antidepressant/anti-anxiety	16 (29.63%)	2 (6.67%)	0.004
Stimulant	9 (16.67%)	2 (6.67%)	0.151

Demographic and psychiatric data for participants in our group analyses; from our initial sample of 85, one proband was excluded due to image quality

^a^Mean and standard deviation are provided for age and full scale IQ. Independent t tests were conducted to determine differences between 22q11DS and control samples

Within the 22q11DS group, 10 participants were currently experiencing positive prodromal symptoms of psychosis (based on a frequency of symptoms > 1 week, and a score of equal or greater than 3 on the positive symptoms subscale of the Structured Interview for Prodromal Symptoms [SIPS; [32]]). An additional 5 participants were diagnosed with overt psychosis. Additional details regarding these subgroups can be found in Table 2. The institutional review board of SUNY Upstate Medical University approved all study procedures, and each participant provided written informed consent or assent.

**Table 2:** Demographic data for prodromal and nonprodromal subgroups

	Prodromal N = 10	Overt N = 5	Nonprodromal N = 39	p value
Age^a^	22.60 (2.50)	19.43 (1.54)	20.76 (2.21)	0.320
Gender (male, %)	5 (50.0%)	2 (40.0%)	23 (58.97%)	0.436
Full scale IQ^a^	71.0 (6.65)	61.6 (4.62)	76.92 (12.53)	0.002

Demographic and psychiatric data for prodromal, nonprodromal, and participants with overt psychosis from our initial sample of 55; 1 proband was excluded due to image quality

^a^Mean and standard deviation are provided for age and full scale IQ. Independent t tests were conducted to determine differences between prodromal and nonprodromal subgroups; participants with overt psychosis were combined with the prodromal group for subsequent analyses

### Psychiatric assessment

Participants had psychiatric evaluations administered by two doctoral-level clinicians (WF and KMA). To determine the presence of DSM-IV psychiatric diagnoses in both the 22q11DS and control group, the Structured Clinical Interview for DSM-IV Axis I disorders (SCID; [33]) was administered. Inter-rater reliability was calculated based on 5 consecutive, audio-recorded interviews resulting in an interclass correlation coefficient of 0.91. The presence of prodromal, positive symptoms of psychosis was determined utilizing the Structured Interview for Prodromal Syndromes (SIPS; [32]), conducted within the context of the psychiatric evaluation. Additional details regarding psychiatric diagnoses can be found in Table 1.

### Image acquisition

Both anatomical and functional resting-state imaging data were acquired with a Siemens Tim Trio, 3 Tesla scanner with an 8-channel head coil receiver (Siemens Medical Solutions, Erlangen, Germany) during the same scanning session. T1-weighted images were acquired in the sagittal plane utilizing a MPRAGE pulse sequence with the following parameters: TR/TE = 2530/3.31 ms, voxel size = 1.0 × 1.0 × 1.0, flip angle = 7^o^, field of view = 256 mm, and 256 × 256 acquisition matrix. Blood oxygen level dependent (BOLD) images were acquired during a 5-minute resting-state scan, which included 152 images (34 axial slices, 4 mm thickness, no gap) utilizing an ep2d_bold sequence: TR/TE = 2000/30 ms, voxel size 4.0 × 4.0 × 4.0, flip angle = 90^o^, field of view = 256, acquisition matrix = 64 × 64. Participants were instructed to keep their eyes open and not to fall asleep during the scanning session.

### Image processing

Raw structural data were imported into the FreeSurfer image analysis suite (v5.1.0, https://surfer.nmr.mgh.harvard.edu/ [34]) for removal of non-brain tissue. The generated brain mask was then manually edited in 3DSlicer 4 (https://www.slicer.org/ [35]). Edited brain masks were then aligned in 3DSlicer along the anterior and posterior commissure using a cubic spline transformation. Resolution was maintained at 1 mm cubic isotropic voxels. Preprocessed data were then introduced into FreeSurfer’s automated surface-based reconstruction and volume-based subcortical processing streams to segment, and parcellate the brain into 68 regions based on the Desikan-Killiany atlas [36]. To briefly summarize, this processing pipeline includes motion correction, intensity normalization, registration to Talairach space, removal of non-brain matter, cortical reconstruction, and segmentation of subcortical structures and white matter. Before final reconstruction was run, manual intervention using control points were placed to minimize motion and hyperintensities that were not corrected by the automated pipeline. Details of manual intervention protocols can be found in McCarthy and colleagues [37]. Second reconstruction was then conducted considering any manual intervention. Final reconstruction steps were then run to complete the processing pipeline.

Functional data were preprocessed using statistical parametric mapping (SPM5; Wellcome Trust Centre for Neuroimaging, 2005, London, UK, http://www.fil.ion.ucl.ac.uk/spm/ [38]). Images were visually inspected for the presence of significant signal dropout, ghosting, excessive noise, and any other artifact that would impact the ability to analyze the images. Visual inspection was repeated throughout different stages of preprocessing. Images were first motion corrected using INRIalign [39], an algorithm that is unbiased by local signal changes. Motion adjustment, an algorithm that suppresses residual fluctuations due to errors in interpolation from large motions was subsequently conducted using ArtRepair [40]. A despiking function was then applied to remove any spikes caused by motion. No participants were excluded due to motion based on the following criteria: > 2 mm across the entire run and rotation greater than 2°. One proband was excluded due to a significant signal dropout in the raw BOLD images, and no other participants were excluded for any other artifacts mentioned above.

Anatomical T1-weighted images from FreeSurfer, (including each ROI for both hemispheres) were then coregistered to the mean functional EPI image in SPM for each participant.

### Functional connectivity analysis

Functional connectivity analyses were conducted utilizing the CONN toolbox (https://www.nitrc.org/projects/conn [28]). This toolbox implements a CompCor method, which reduces physiological and movement effects: CSF and white matter effects, task-related effects, and realignment parameter noise without removing the global signal [29]. A band-pass filter of 0.008–0.09 was applied to the data. Realignment parameters from preprocessing were entered as confounds in the first-level analysis. Using the Desikan-Killany atlas in FreeSurfer [36], which generates 34 bilateral, or 68 ROI’s, we conducted a seed-based ROI-to-ROI analysis to create a 68 × 68 functional connectivity map. A bivariate correlation was used to determine total linear temporal associations between each of the resulting 2278 ROI-to-ROI functional connections. Second-level analyses of group differences in functional connectivity between 22q11DS and controls was conducted through the CONN toolbox and FDR-corrected, *p* < 0.05, two-tailed.

We then repeated the aforementioned ROI-to-ROI analysis to compare functional connectivity between prodromal and nonprodromal participants with 22q11DS based on positive symptoms that were present at a frequency of greater than once per week, and that obtained summed scores of ≥ 3 (reflecting intensity of the symptom) on the Structured Interview for Prodromal Symptoms (SIPS; [32]) positive symptoms subscale. These criteria have been applied in previous studies of individuals with 22q11DS [20, 24].

### Associations with positive symptoms

We then tested associations between positive symptom scores in 22q11DS (taken from summed scores of the SIPS Positive Symptoms subscale) and functional connectivity values for ROI-to-ROI connections that were significantly different between individuals with 22q11DS and the control group. Functional connectivity values were taken from Fisher-transformed correlation coefficients from the first-level analysis conducted in the CONN toolbox. Since many participants with 22q11DS scored 0 on the SIPS Positive Symptoms Scale (29 participants, 53.7%), and since the SIPS produces a count variable, we conducted a zero-inflated Poisson (ZIP) regression analysis to examine these associations. Results were then FDR-corrected, *p* < *0.05.*

## Results

**Fig. 1:**
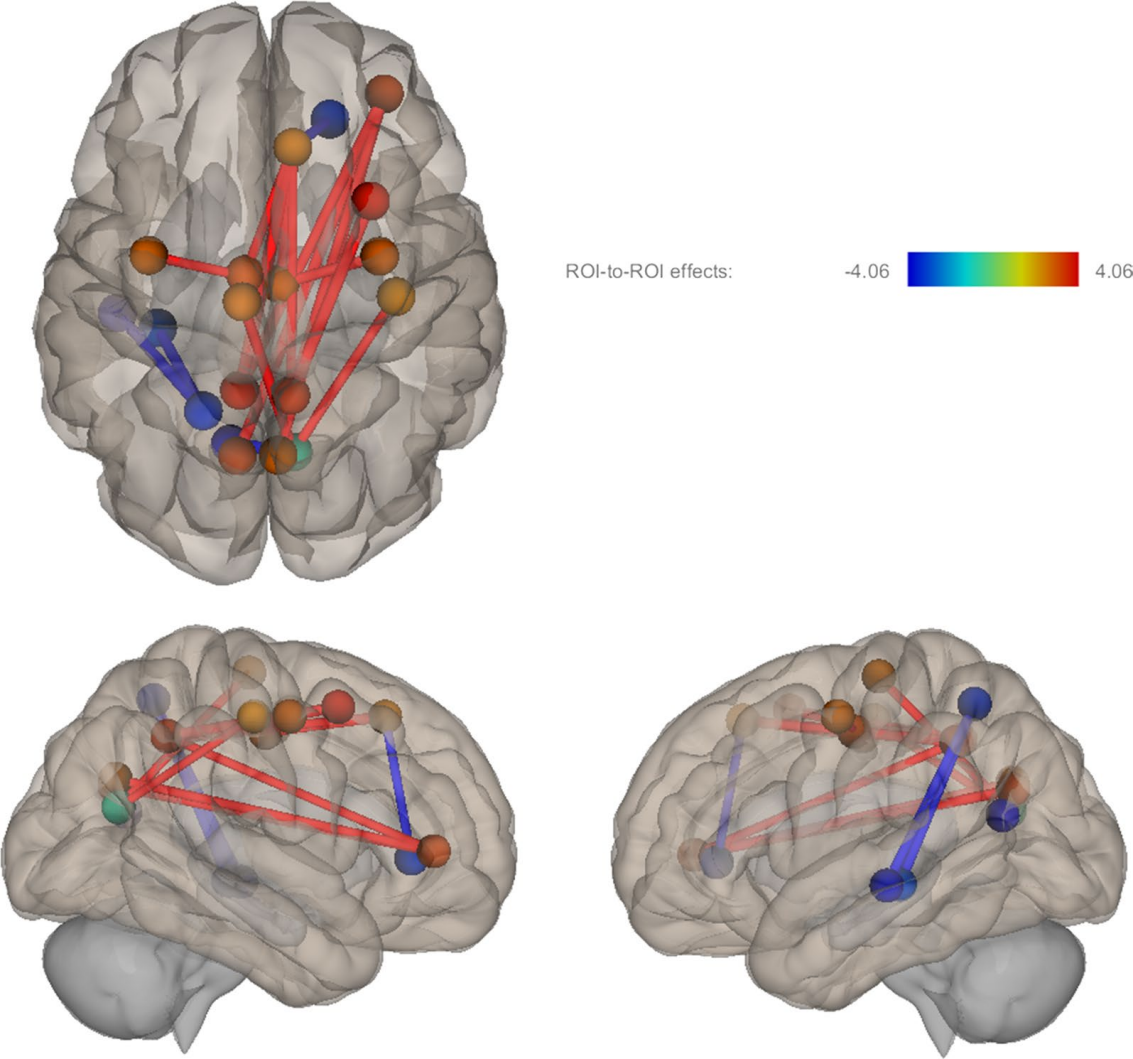
This figure depicts significant differences in functional connectivity between 22q11DS and control samples. The color bar represents t values of results in axial (top) and left and right sagittal views. Red indicates increased FC in 22q11DS and blue indicates reduced FC in 22q11DS

Second-level analyses of the functional connectome analysis revealed significant differences in functional connectivity between 22q11DS and controls (p_FDR_ < 0.05). (Table 3 and Fig. 1) At the lobar level, we observed differential connectivity between ROIs within frontal–frontal, frontal–occipital, frontal–parietal, occipital–occipital, and parietal–temporal regions.

**Table 3:** Differential functional connectivity between 22q11DS and controls

Functional connection (ROI–ROI) 22q11DS vs controls	Lobar-level connections	t value	p value, corr	22q11DS^a^	controls^a^
Right precentral–right posterior cingulate	Frontal–frontal	3.59	0.038	0.232	0.067
Right superior frontal–left posterior cingulate	Frontal–frontal	3.22	0.025	0.230	0.036
Right superior frontal–right posterior cingulate	Frontal–frontal	3.23	0.025	0.411	0.212
Right pars orbitalis–left cuneus	Frontal–occipital	3.79	0.019	0.011	− 0.187
Right pars orbitalis––right cuneus	Frontal–occipital	3.44	0.022	0.021	− 0.146
Right pericalcarine–left paracentral	Frontal–occipital	3.42	0.033	− 0.013	− 0.173
Right pericalcarine–right postcentral	Frontal–occipital	3.27	0.035	− 0.013	− 0.159
Right precuneus–right caudal middle frontal	Frontal–parietal	4.04	0.008	0.281	0.054
Left Precuneus–right pars orbitalis	Frontal–parietal	3.42	0.033	− 0.109	− 0.313
Right precuneus–right pars orbitalis	Frontal–parietal	3.23	0.04	0.014	− 0.174
Left precuneus–right superior frontal	Frontal–parietal	4.06	0.008	0.110	− 0.113
Right precuneus–right superior frontal	Frontal–parietal	3.30	0.04	0.289	0.092
Right superior frontal–right lateral orbito frontal gyrus	Frontal–frontal	− 3.37	0.025	0.102	0.312
Right pericalcarine–left pericalcarine	Occipital–occipital	− 3.98	0.01	1.254	1.488
Left superior parietal–left fusiform	Parietal–temporal	− 3.55	0.021	0.208	0.382
Left superior parietal–left inferior temporal gyrus	Parietal–temporal	− 3.63	0.021	0.156	0.379

Functional connections displayed within this table represent connections that were significantly different between 22q11DS and controls, FDR-corrected, *p* < *0.05*

^a^Mean functional connectivity values reported for each study group

### Increased functional connectivity in 22Q11DS vs. controls

Within frontal–frontal connections, we observed increased functional connectivity in individuals with 22q11DS relative to controls between the right precentral gyrus and right posterior cingulate, right superior frontal gyrus to left posterior cingulate, and right superior frontal gyrus to right posterior cingulate. Table 3 displays differential functional connections between 22q11DS and controls at both the lobar and sublobar level as well as t values, corrected p values, and averaged functional connectivity values.

Increased functional connectivity was also observed in frontal–occipital connections: between the right pars orbitalis and left cuneus, right pars orbitalis and right cuneus, right pericalcarine and left paracentral gyri, and right pericalcarine and right postcentral gyri. Relative to controls, increased functional connectivity was again displayed within frontal-parietal connections: between the right precuneus to the right caudal middle frontal gyrus, left precuneus and right pars orbitalis, right precuneus and right pars orbitalis, left precuneus and right superior frontal gyrus, right precuneus and right superior frontal gyrus.

### Reduced functional connectivity in 22Q11DS vs. controls

Reduced functional connectivity was observed between the right superior frontal gyrus and right lateral orbitofrontal cortex. We also observed reduced functional connectivity in 22q11DS in parietal-temporal connections: between the left superior parietal lobule and left fusiform gyrus and left superior parietal lobule and left inferior temporal lobe.

### Functional connectivity within 22Q11DS

**Fig. 2:**
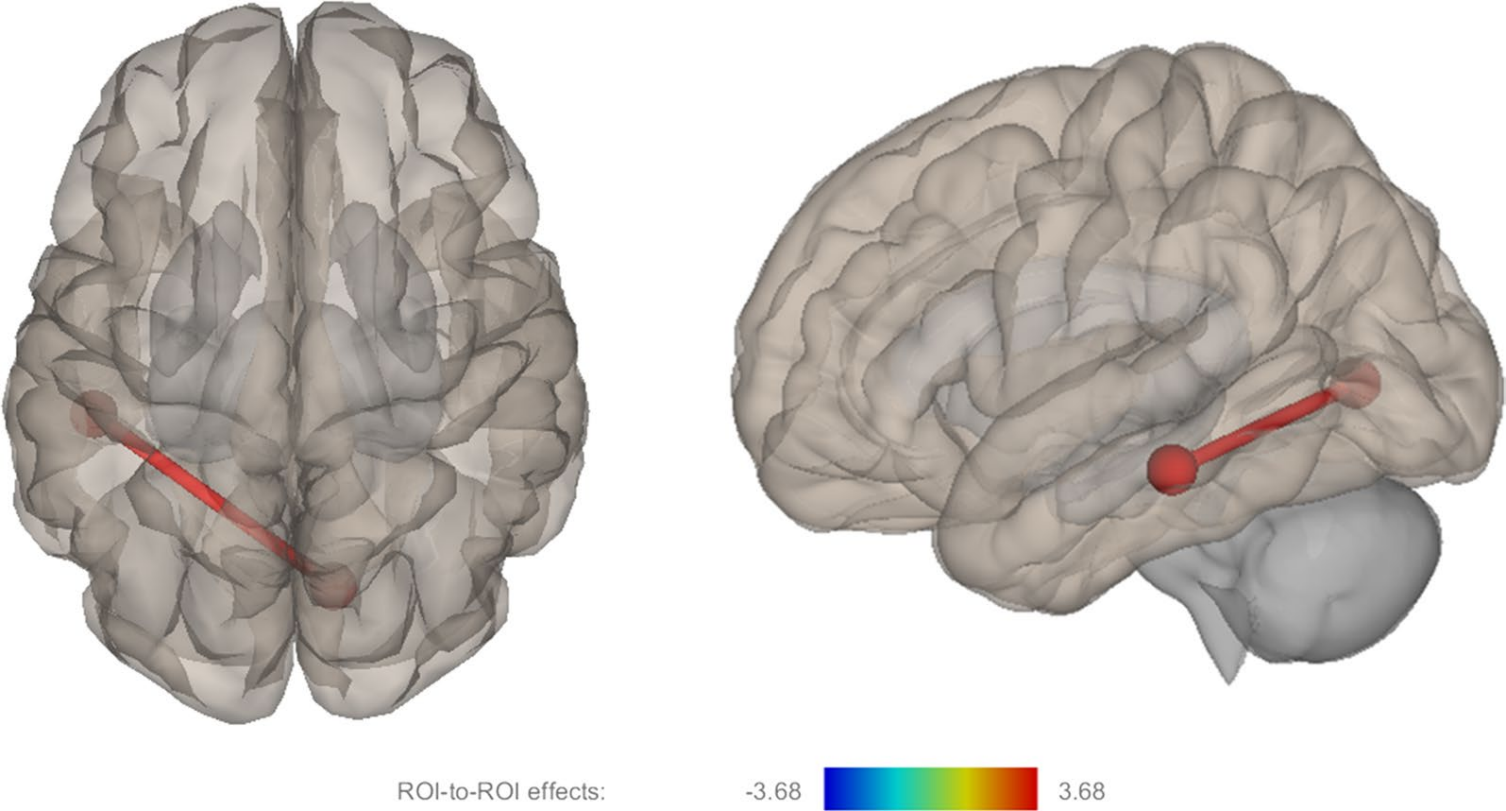
This figure depicts differential functional connectivity between prodromal and nonprodromal (prodromal > nonprodromal) 22q11DS samples represented by left sagittal and superior axial views

Between the nonprodromal and prodromal 22q11DS groups, we observed only one significant difference between groups: increased functional connectivity between the left inferior temporal and right pericalcarine gyri (t = 3.68, p_FDR_ = 0.038) (Fig. 2).

### Associations with psychosis in 22q11DS

**Fig. 3:**
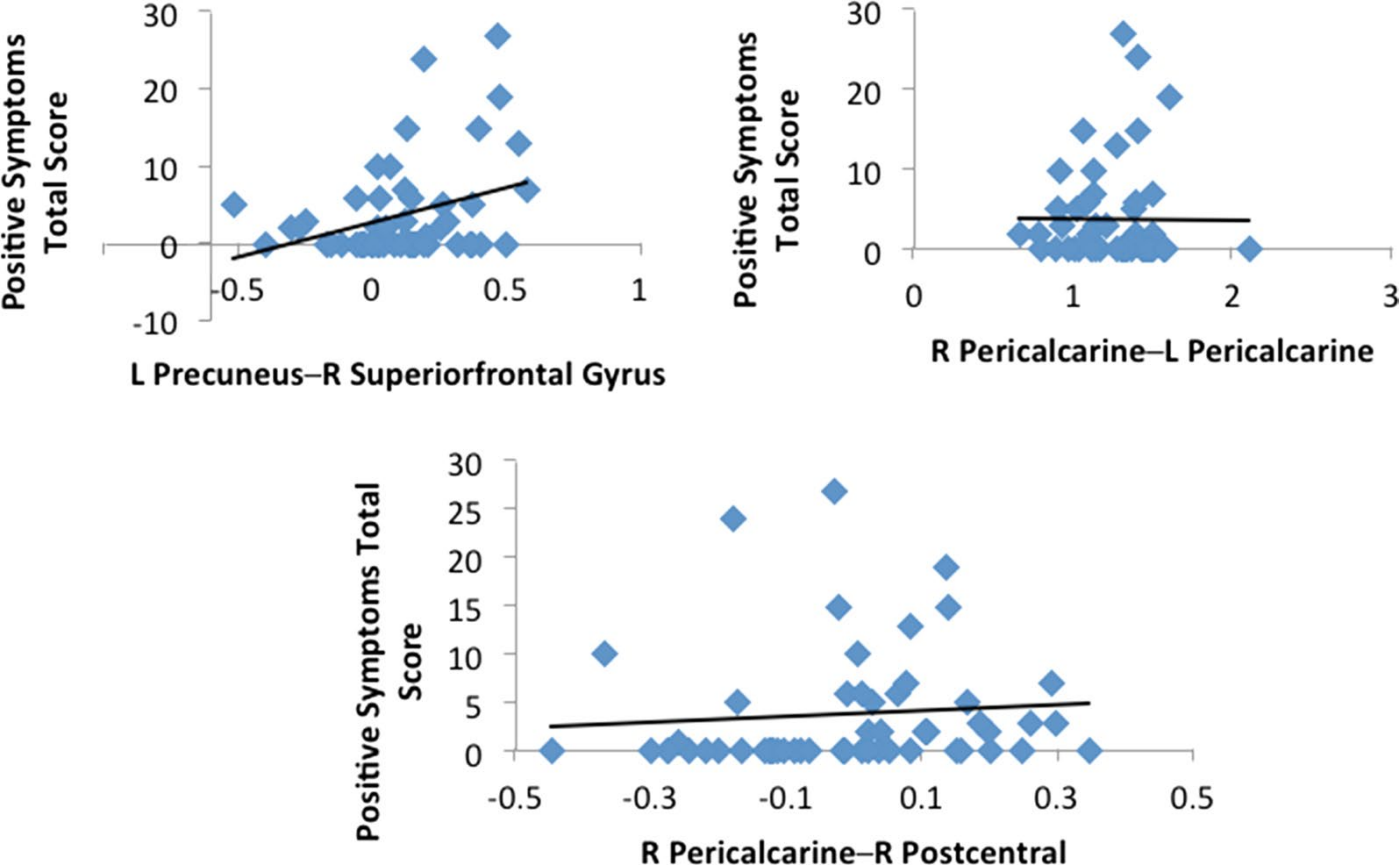
This figure depicts plots representing associations between total positive symptoms scores measured by the SIPS and functional connectivity in connections that were significantly different between 22q11DS and controls

After correction for multiple comparisons, (p_FDR_ < 0.05) a ZIP regression analysis reported increased functional connectivity between the left precuneus and right superior frontal was positively associated with positive symptoms (z = 5.72, *p* = 0.008). Reduced functional connectivity between the right pericalcarine and left pericalcarine was positively associated with positive symptoms (z = 4.39, *p* = 0.008). Increased functional connectivity between the right pericalcarine and right postcentral were found to be negatively associated with positive psychotic symptoms (z = − 2.95, *p* = 0.016) (see Fig. 3).

### Heterogeneity effects in controls

Since seven of our controls in the current report were diagnosed with an anxiety disorder, depression, or ADHD, we conducted a separate functional connectivity analysis in CONN excluding those seven participants to account for any potential confounding effects in our FC results. Our findings remained significant after FDR correction, *p* < 0.05, and we continued to observe the same patterns of increased/decreased functional connectivity between the frontal–occipital, frontal–parietal, occipital–occipital, and superior parietal-inferior temporal connections. However, we did observe that once these controls were excluded, functional connectivity between frontal–frontal regions (superior frontal lobe–posterior cingulum; precentral gyrus–posterior cingulum) and one frontal–parietal connection (pars orbitalis–precuneus) no longer met threshold for significance.

## Discussion

Using a seed-based connectivity analysis of 2278 ROI-to-ROI connections, we observed both hyper- and hypo-connectivity in frontal–frontal gyri, frontal–parietal gyri, frontal–occipital gyri, parietal–temporal gyri and occipital–occipital gyri in young adults with 22q11DS relative to controls. Notable findings included (1) increased functional connectivity between frontal (superior frontal, caudal middle frontal and pars orbitalis) gyri and the precuneus, and (2) increased functional connectivity between posterior cingulate gyrus and both superior frontal and precentral gyri. Anomalies in frontal–parietal and occipital–occipital gyral connectivity were significantly associated with positive symptoms of psychosis.

The precuneus, caudal middle frontal and pars orbitalis (i.e., medial inferior frontal) regions constitute part of the default mode network (DMN), which as noted above, is reported to be anomalous in both schizophrenia and 22q11DS. Studies have demonstrated that the DMN is active not only during rest but also during activities involving self-referential [41] and social-interpersonal processing [42]. Evidence suggests that the DMN may be involved in auditory hallucinations in individuals with schizophrenia [43–45], although other networks have been implicated as well [46, 47]. In individuals with 22q11DS, the DMN has been associated with prodromal symptoms [21], sustained attention [21] and reciprocal social behaviors [23]. It is not clear why we observed increased functional connectivity between these DMN regions, while several other studies [23–26] of 22q11DS have observed decreased functional connectivity between these regions. This may be attributable, in part, to our implementation of measurements within each subject’s native brain space. In light of the anatomic differences that have been reported in brains of individuals with 22q11Ds, retaining each subject’s native brain space may have produced results that are not totally (anatomically) comparable to studies in which brains are warped to a standard template. Moreover, potential differences in sample characteristics (e.g. IQ levels; medication usage) between studies may also be contributing to differences in the direction of these results (see review by Scariati and colleagues [19]). Additional insight into why our finding of increased functional connectivity in the DMN differs from several (but not all [21, 22]) studies of 22q11DS is suggested by the results of two previously-published papers [22, 24] that included samples that overlapped with the sample of the current. In our two previously-published papers, we pooled samples from two research sites, and applied Independent Components Analyses to the pooled data. However, preprocessing methods differed somewhat between the two papers. In the first paper, by Mattiaccio and colleagues [22], for which data were preprocessed and analyzed at our site, increased functional connectivity in the DMN was observed. In the second paper, by Schreiner and colleagues [24], the data were preprocessed and analyzed by our collaborating site, and decreases in functional connectivity in the DMN were observed. Interestingly, our respective sites’ preprocessing methods differed in motion correction and noise reduction strategies, potentially accounting for the discrepancies in results. This supports the notion that differences in image processing methods and in sample characteristics may be contributing to between-study differences in results.

The posterior cingulate gyrus (PCG) is also part of the default mode network, and we found anomalies in connectivity between PCG and superior frontal and precentral gyri. The extent to which PCG—superior frontal connections in our study reflects the DMN is not completely clear, since we utilized a predefined, atlas-based approach that maps onto regions that subsume, but are not synonymous with the DMN. Nonetheless, primate (and more recently, human imaging) studies indicate that the PCG has strong, reciprocal connections to the dorsolateral prefrontal cortex (DLPFC) [48–50], which overlaps with the superior frontal region included in the Desikan-Killany atlas. It has been suggested that PCG-DLPFC connections may be part of both the dorsal attention network and the frontal-parietal control network [51] both of which contribute to efficient cognitive function. Functional connectivity of the PCG and the superior aspect of the DLPFC has been linked to goal-directed thought processes [52], suggesting that this reciprocal connection may subserve executive planning [53, 54] and cognitive control [53, 55], both of which are impaired in individuals with 22q11DS [56–59]. Moreover, these functional brain networks have been shown to be impaired in schizophrenia [14, 60, 61] and 22q11DS [22, 24, 62].

Of the 16 ROI-to-ROI connections that significantly differentiated individuals with 22q11DS from controls, 13 (81%) of them included at least one ROI in the frontal lobe. These findings are consistent with other functional connectivity studies of both idiopathic schizophrenia [7, 12, 13, 63] and 22q11DS [20, 23] and suggest that both short-range and long-range connectivity of the frontal lobe is anomalous in individuals with this syndrome. To the extent that the frontal lobe subserves a myriad of cognitive and social-affective functions, functional dysconnectivity of networks that include the frontal lobe could underlie many of the cognitive and psychiatric impairments that are associated with 22q11DS [20, 23]. For example, in addition to schizophrenia, frontal dysconnectivity has been implicated in both autism spectrum disorders and in ADHD, both of which are elevated in 22q11DS [5, 57, 64–68].

In our sample, positive prodromal symptoms of psychosis were associated with increased connectivity between the superior frontal gyrus and the precuneus, and with decreased connectivity between the right and left pericalcarine gyri of the occipital lobe, and between pericalcarine and postcentral gyri. As noted above, the precuneus and aspects of the superior frontal gyrus are included in the DMN, which previous studies of 22q11DS have associated prodromal symptoms as well [21]. Associations between parietal–occipital and occipital–occipital functional connections and prodromal symptoms of psychosis have not been reported. However, anatomic connections between parietal and occipital lobes, via the superior longitudinal fasciculus (SLF), have been reported to be aberrant in 22q11DS [69–72]. Moreover, in an overlapping sample, our group [73] recently reported associations between anatomic anomalies in the SLF and prodromal symptoms.

When we divided the group of individuals with 22q11DS into prodromal and nonprodromal subgroups, we observed a significant difference in connectivity between the left inferior temporal and right pericalcarine gyri. Interestingly, we recently reported (in the same patient sample) significant associations between white matter microstructural anomalies in the temporal-occipital aspect of the inferior longitudinal fasciculus and symptoms of psychosis [74]. Temporal-occipital alterations in functional connectivity have also been reported in patients experiencing their first episode of psychosis [75], further supporting the validity of these observations.

## Limitations and conclusions

Our study utilized an atlas-based approach to investigate functional connectivity in 22q11DS, which permitted us to examine, within each individual’s own fMRI space, more than 2000 functional connections throughout the cortex. A potential limitation to our method is that the acquisition time of 5 min that we used to acquire our fMRI data, while minimally acceptable for an fcMRI study, may not be optimal in order to minimize the effects of noise and ensure the detection of small correlations that might otherwise go unobserved [76]. A second potential limitation is that the connections we examined do not necessarily map specifically onto the networks that are traditionally examined in resting state fcMRI studies, thus limiting comparisons to other studies to some extent, and rendering conclusions regarding these comparisons somewhat speculative. Nonetheless, our results concur generally with previous studies that have observed DMN anomalies in 22q11DS and associations between DMN anomalies and prodromal symptoms of psychosis. However, we observed increased functional connectivity in DMN regions, in contrast to several previous studies that have observed reduced connectivity. As noted above, this may be due in part to the potential impact of current medication usage in our sample, and to study differences in image preprocessing. In addition, it should be noted that when we removed the subset of controls with ADHD and anxiety, study group differences in the connections between the PCG and both the superior frontal and precentral gyri did not survive correction for multiple comparisons. This may suggest that the presence of psychiatric disorders in our sample may be influencing our observation of study group differences in connectivity between PCG and other frontal-based regions; however, the removal of the control subgroup also reduced power to detect differences. Accordingly, future studies would benefit from larger samples to elucidate the potential interplay between the presence of psychiatric disorders in 22q11DS and functional connectivity. To the extent that sampling and image preprocessing differences account for discrepancies across studies, it would be useful, in general, to apply different preprocessing methods to identical samples in order to elucidate the extent to which these methods account for differences in results of functional connectivity studies. Within the area of neurofunction in 22q11DS, future studies should examine the associations between functional and structural connectivity in 22q11DS, in order to elucidate the extent to which neuroanatomic structure underlies functional anomalies and leads to the psychiatric impairments for which individuals with this disorder are at great risk.

## Additional file

**Additional file 1.** Additional material.

## Abbreviations

22q11DS: 22q11.2 deletion syndrome
FC: functional connectivity
ROI: region of interest
SCID: Structured Clinical Interview for DSM-IV Axis I disorders
SIPS: Structured Interview for Prodromal Symptoms
SPM: statistical parametric mapping
DMN: default mode network
PCG: posterior cingulate gyrus
DLPFC: dorsolateral prefrontal cortex
ADHD: attention deficit hyperactivity disorder
SLF: superior longitudinal fasciculus
corr: corrected
