# Supplementary material for: Frontal dysconnectivity in 22q11.2 deletion syndrome: an atlas-based functional connectivity analysis
Source: Behav Brain Funct. 2018 Jan 20;14:2. doi: 10.1186/s12993-018-0134-y (PMC5775582; doi:10.1186/s12993-018-0134-y)
Supplement: Supplementary file 1 — Additional file 1. Additional material. [file 12993_2018_134_MOESM1_ESM.docx]

Supplementary Material: Initial analyses comparing functional connectivity between siblings and community controls.

To account for any possible confounding effects by combining siblings and community controls into a single group for comparison with 22q11DS patients, we conducted another functional connectivity analysis utilizing the CONN toolbox with the same model parameters described previously testing differential connectivity between siblings and community controls. Testing all 68 ROI-ROI functional connections, we observed significant differences (p_FDR_ <0.05) between only two connections: the left inferior temporal gyrus and the right superior temporal gyrus, and the left inferior temporal gyrus and right middle temporal gyrus. Since only two functional connections were found to be significant after correction for multiple comparisons, we combined the sibling and community control groups into a single control group for the present report.

Supplementary Fig. 1 This figure depicts functional connectivity values for siblings and community controls, for the connections that differed significantly between the two groups.
